# Supplementary material for: Genetic and Pathogenic Variability of Mycogone perniciosa Isolates Causing Wet Bubble Disease on Agaricus bisporus in China
Source: Pathogens. 2019 Oct 8;8(4):179. doi: 10.3390/pathogens8040179 (PMC6963780; doi:10.3390/pathogens8040179)
Supplement: Supplementary file 1 [file pathogens-08-00179-s001.pdf]

Supplementary Table 1: Conidia production (conidia per mL) of 18 isolates of *Mycogone perniciosa* growing on PDA medium at 25°C for 7 days.

| No | Isolates | **Concentration of conidia production**<br>**Conidia/ mL**<br>**($10^6$)** |
|---|---|---|
| 1 | Hp1 | 0.003 ±0.001[a] |
| 2 | Hp2 | 0.000 |
| 3 | Hp3 | 0.002 ±0.001[a] |
| 4 | Hp4 | 0.006 ±0.002[a] |
| 5 | Hp5 | 0.067 ±0.029[a] |
| 6 | Hp6 | 0.233 ±0.029[a] |
| 7 | Hp7 | 0.467 ±0.029[a] |
| 8 | Hp8 | 0.000 |
| 9 | Hp9 | 0.037 ±0.046[ab] |
| 10 | Hp10 | 0.517 ±0.076[a] |
| 11 | Hp11 | 0.317 ±0.029[a] |
| 12 | Hp12 | 0.283 ±0.029[a] |
| 13 | Hp13 | 0.02 ±0.003[a] |
| 14 | Hp14 | 0.03 ±0.004[a] |
| 15 | Hp15 | 0.067 ±0.029[a] |
| 16 | Hp16 | 0.067 ±0.058[a] |
| 17 | Hp17 | 0.10 ±0.050[a] |
| 18 | Hp18 | 0.60 ±0.180[a] |

Results followed by the same letter in the column do not differ significantly (Duncan's Multiple Range Test, $P < 0.05$). *$P = 0.0001$
